# An Effective Method of *Ribes* spp. Inoculation with Blackcurrant Reversion Virus under In Vitro Conditions

**DOI:** 10.3390/plants11131635

**Published:** 2022-06-21

**Authors:** Ana Dovilė Juškytė, Ingrida Mažeikienė, Vidmantas Stanys

**Affiliations:** Lithuanian Research Centre for Agriculture and Forestry, Institute of Horticulture, Kaunas str. 30, 54333 Babtai, Lithuania; ingrida.mazeikiene@lammc.lt (I.M.); vidmantas.stanys@lammc.lt (V.S.)

**Keywords:** BRV, in vitro inoculation method, transmission through roots, host resistance, *Ribes* spp.

## Abstract

Blackcurrant reversion virus (BRV) is the most destructive currant-infecting and mite-transmitted pathogen from the genus *Nepovirus*. In this work, BRV transmission in the system *Ribes* ex vitro–*Ribes* in vitro was applied for the first time. Triple infection of BRV identified in blackcurrant cv. Gojai was used for phylogenetic analysis and inoculation assay. Transmission of BRV was successful due to its stability in the inoculum for up to 8 days at 4 °C; all BRV isolates were infectious. Our suggested inoculation method through roots was applied in six *Ribes* spp. genotypes with 100.0% reliability, and the expression levels of defence-related gene *PR1* to biotic stress was observed. The prevalence of the virus in microshoots after 2–14 days post-inoculation (dpi) was established by PCR. In resistant genotypes, the BRV was identified up to 8 dpi; meanwhile, infection remained constant in susceptible genotypes. We established that BRV transmission under controlled conditions depends on the inoculum quality, post-inoculation cultivation temperature, and host-plant susceptibility to pathogen. This in vitro inoculation method opens possibilities to reveal the resistance mechanisms or response pathways to BRV and can be used for the selection of resistant *Ribes* spp. in breeding programs.

## 1. Introduction

Blackcurrant reversion virus (BRV) is transmitted by a specific biological vector—eriophyid mites from *Cecidophyopsis* spp. (Acari: *Eriophyidae*). The virus is the causative agent of blackcurrant reversion disease (BRD). The pathogen (BRV), vector (*C. ribis*), and disease (BRD) complex causes significant yield losses in blackcurrant plantations worldwide [1]. *R. nigrum* L. is the primary natural host of BRV, although natural infestations also occur in other species—*R. pauciflorum*, *R. rubrum*, *R. alpinum*, *R. aureum*, etc. One of the best ways to reduce BRV economical losses is the targeted selection of genetically resistant cultivars [2]. Species *R. uva-crispa*, *R. cereum*, *R. dikuscha*, *R. nigrum* var. *sibiricum*, etc., have resistance determined by genes to gall mite and consequently to BRV [3,4,5,6]. However, only several molecular markers of resistance genes are known and can be used for marker-assisted selection (MAS) in blackcurrant breeding programs [7,8,9].

The BRV belongs to the subgroup c of the genus *Nepovirus*, family *Secoviridae* [10,11]. Virus isometric particles are approximately 27 nm in diameter, and the viral genome consists of two polyadenylated, single-stranded positive-sense RNA1 and RNA2 molecules [12,13]. It is known that the 3′ nontranslated region (NTR) of RNAs are essential for virus lifecycles, promoting translation regulation and its efficiency [14], and consequently were used for BRV detection or phylogenetic analysis [6,15,16,17].

The BRD according to symptoms is divided into two forms—European (E) and Russian (R) [4,18]. The main difference between the forms is the severity and rate of symptoms expressed in the infected plants. Both forms cause a transformation in leaf shape, a decrease in marginal serration and in the number of main veins and hair numbers, and an increase in colour intensity on the flower buds. However, the flower sterility resulting in a complete loss of berry yield occurs only in the R form of reversion disease [4,5,6,7,8,9,10,11,12,13,14,15,16,17,18,19].

Atypical to other *Nepovirus*, BRV is not transmitted through seeds or pollen. The BRV, the biological vector, and the host plant have a specific relationship [20]. Mechanical inoculations using sap extracts are usually complicated and fail by the very low concentration of virus particles, their erratic distribution in plants, and the rapid inactivation of the virus by phenolic compounds in leaves of *Ribes* plants [10,19,21]. BRV transmission in vivo is possible by the graft inoculation method in *R. nigrum* [18,22,23]. In this case, reversion symptoms on plants occur after 2–4 vegetation seasons [20,23]. This inoculation technique in field or greenhouse conditions is not suitable for rapid and sensitive biotechnological and genetic research. A more reliable inoculation method of BRV for biotechnological purposes would be under in vitro conditions, but such research is limited. No scientific data about successful cases of BRV inoculation in the *Ribes* to *Ribes* system are available.

Inoculation of plants in vitro would offer the potential for great savings in time and for efficient experimentation under controlled conditions. It is an accurate tool to study the epidemiology of BRD and the interrelationships between the virus and the host at molecular and genetic levels. Thus, the aim of this study was to develop a rapid and efficient inoculation method for BRV under in vitro conditions, emphasizing genetic diversity, stability, and infectivity of BRV.

## 2. Results

### 2.1. Source of BRV for Inoculation In Vitro

Heterogeneous infection of BRV in the same plant of Lithuanian blackcurrant cv. Gojai was detected. This was proved by the multiple alignment analysis of 3′ NTR RNA2 sequences. Three different BRV isolates with various mutations were found: BRV_1-18_LT, BRV_7-18_LT, and BRV_3-18_LT. Virus isolates identified in this study were uploaded into the NCBI database with accession numbers MH891843, MH891844, and MH891845. Genetic identity among isolates ranges from 94.6 to 99.6% in the 3′ nontranslated region of RNA2 (Table 1).

**Figure 1:**
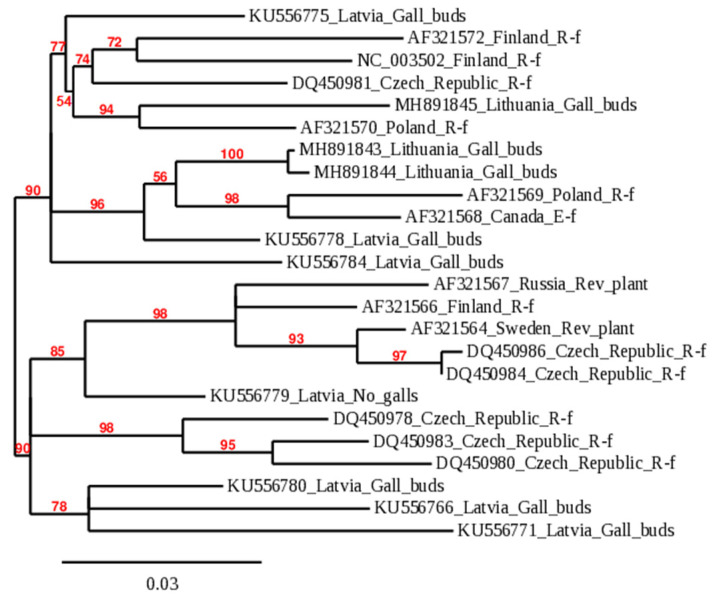
Genetic relationship among worldwide isolates of BRV according to 3′ NTR RNA2 sequences. The label of members in the phylogenetic tree consists of accession number in NCBI_country of origin_blackcurrant reversion disease form or symptom. Accession numbers of BRV sequences obtained in this research are MH891843–MH891845; characteristics of isolates of other plants shown in Figure 1 are available in Table S2 in Supplementary Materials.

A phylogenetic dendrogram was constructed to determine the viral infection affinity using sequences of BRV identified in Lithuania and sequences submitted to the NCBI database (Figure 1). Worldwide isolates of BRV formed two reliably distinct branches at 90.0% bootstrap. The tree provides information on the geographic origin of the isolates and the implicit migration of infection. According to the symptoms of the disease caused in host plants, virus isolates in the phylogenetic tree were classified as the R (Russian) form of BRD in most cases, except for the isolate from Canada or plants with virus symptoms. All isolates from Lithuania were grouped into the phylogenetic dendrogram’s first branch. In this branch, BRV isolates were still reliably separated into several groups, and BRV_1_18_LT and BRV_7_18_LT formed a separate phylogenetic branch at 100.0% bootstrap. These sequences of RNA2 3′ NTR were genetically close to each other but significantly genetically diverse from other BRV isolates worldwide. Isolate BRV_3_18_LT showed diversity from BRV_1_18_LT and BRV_7_18_LT but was genetically close to an isolate from Poland (AF321570).

### 2.2. Reliability of the Inoculation In Vitro

**Figure 2:**
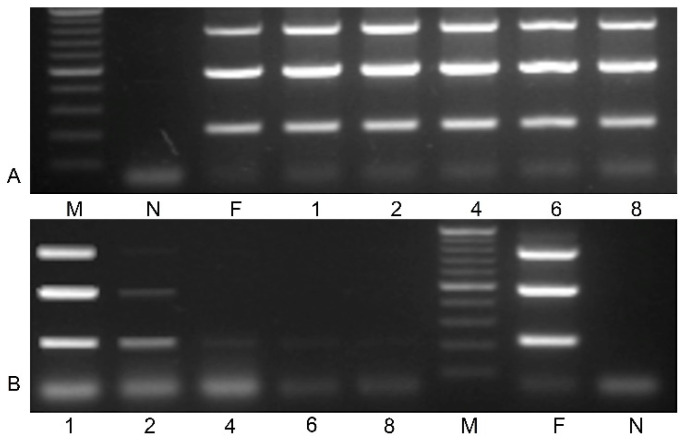
Stability of virus RNA2 in inoculum at 4 °C (**A**) and 21 °C (**B**) storage temperature (M—gene ruler 100–1000 bp; N—negative control; F—fresh inoculum; inoculum after storage 1–8 days. PCR mix without cDNA was used as a negative control.

Symptomatic leaves of blackcurrant cv. Gojai containing heterogeneous infection of BRV, obtained from the plant genetic resources collection of LAMMC, were used in the preparation of inoculum. Stability of RNA2 of BRV in inoculum was investigated. Prepared inoculum was maintained at 4 and 21 °C for up to 8 days. Specific primer pairs (Table 2) for RNA2 were used for BRV identification. Amplification results of BRV-specific fragments in inoculum during different storage temperatures are presented in Figure 2. Virus RNA2 was detected in inoculum at 4 °C during the entire period of the experiment (Figure 2A), while BRV-specific fragments were amplified and clearly shown in the inoculum only after the first day in storage at 21 °C (Figure 2B).

**Figure 3:**
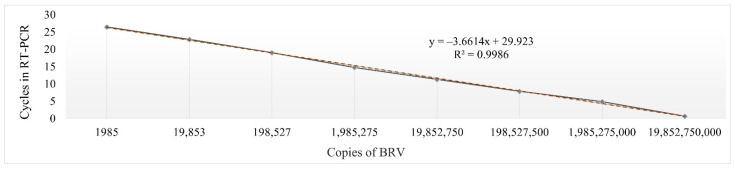
The standard curve of RT-PCR detecting BRV. The plasmids were used to establish the standard curve and the software Microsoft Excel was used to analyse the relationship of Ct values and plasmid copy numbers.

The standard curve for quantification of the virus concentration by RT-PCR with eight points was constructed (Figure 3). Plasmid vector with RNA2 3′ NTR of the BRV was used to determine the number of copies of the virus. Through the range from 1.98 × 10^3^ to 1.98 × 10^10^ copies per reaction, the threshold cycle (Ct) and copy numbers displayed a linear relationship with an R^2^ of 0.9986 and the equation y = –3.6614x + 29.923 (Figure 3). The value of Ct obtained in the inoculum was 21.73 (±0.15).

**Figure 4:**
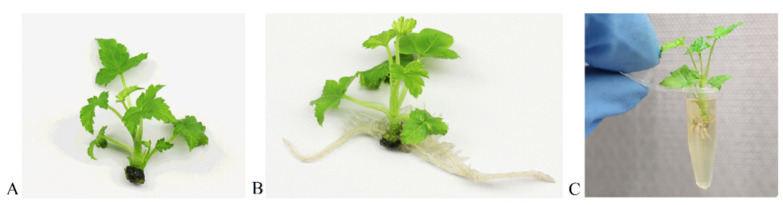
In vitro inoculation of *R. nigrum* microshoots: (**A**) microshoot before rooting; (**B**) microshoot with roots; (**C**) microshoot soaked in the tube with inoculum.

To determine the efficiency of the inoculation method in vitro, microshoots with roots of *R. aureum*, *R. dikuscha*, and *R. nigrum* cvs. Aldoniai, Ben Gairn, Ben Tirran, and Vernisaz were inoculated by soaking in the same way as shown in Figure 4. The roots increase the contact area between the inoculum and the plant, and enhance the entry of the virus into the phloem. Based on our data in Figure 2, a post-inoculation period at 4 °C is necessary to ensure viability of the virus on the surface of the roots.

**Figure 5:**
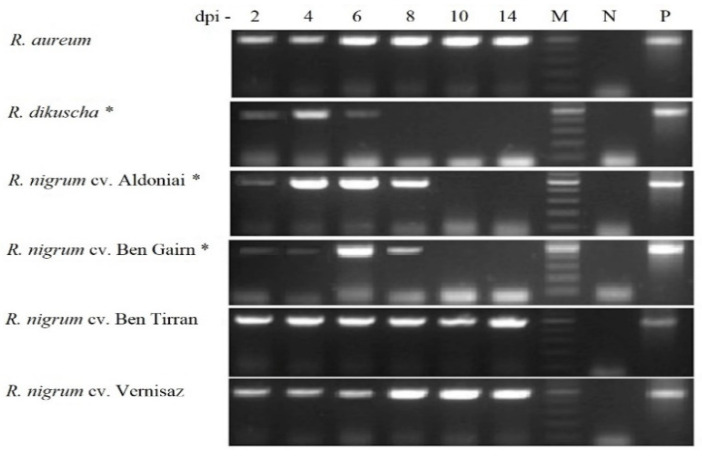
PCR product of the BRV (481 bp) in *Ribes* microshoots after 2–14 dpi. M—gene ruler 100–500 bp; N—negative controls were virus free plants; P—positive control was inoculum; *—BRV-resistant genotypes.

In the post-inoculation period at 2, 4, 6, 8, 10, and 14 days, the virus infection in microshoots without roots was evaluated by PCR (Figure 5). According to the data for 481 bp-length BRV-specific fragment sequencing, all isolates of BRV were transmitted to infected plants by in vitro mechanical inoculation. The nucleotide sequences of these fragments completely coincided with the sequence provided to NCBI (MH891843, MH891844, and MH891845). This indicates that BRV strains obtained in the cultivar Gojai have the potential to contaminate and spread in an in vitro system.

**Figure 6:**
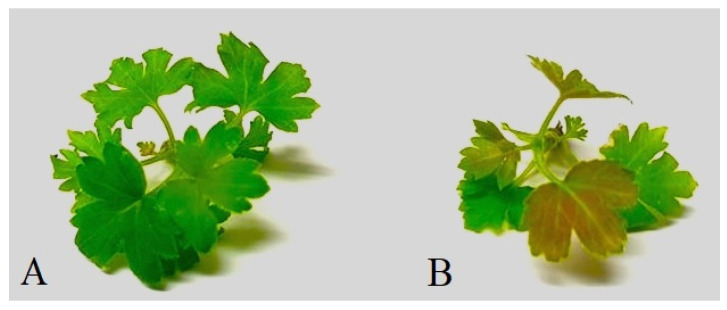
Microshoots of *R. aureum* after 6 dpi: (**A**) BRV-free mock-inoculated microshoot; (**B**) BRV-infected microshoot.

In vitro plants of *R. dikuscha* and *R. nigrum* cvs. Aldoniai or Ben Gairn remained symptom-free in the post-inoculation period. Clearly, symptoms of response to biotic stress were visible on *R. aureum* microplants after 6 dpi (Figure 6). The leaves changed their colour, and the clear synthesis of the stress-characteristic phenolic compounds became visible.

The incidence rate of BRV in all *Ribes* cultivars and species was 100% for 20 microshoots in each treatment. However, the persistence of the infection in the plant was different. Until the end of the experiment, 2 weeks after inoculation, the infection remained constant in the BRV-susceptible genotypes: *R. aureum* and *R. nigrum* cvs. Ben Tirran and Vernisaz. The viral infection was detected in the resistant plants, *R. dikuscha* and *R. nigrum* cv. Ben Gairn, until 6 dpi, and in *R. nigrum* cv. Aldoniai until 8 days after infection (Figure 5).

### 2.3. Relative Gene Expression in Response to BRV Infection

**Figure 7:**
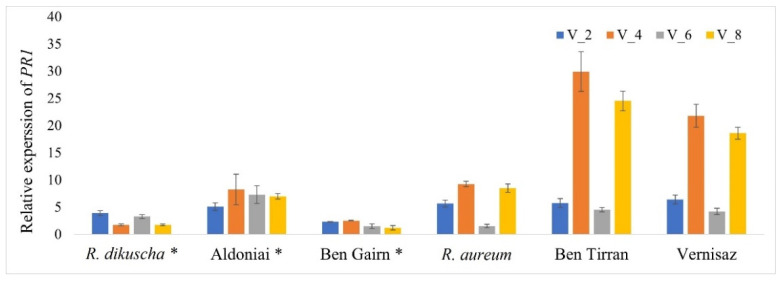
Relative expression levels of *PR1* in resistant and susceptible *Ribes* genotypes in response to BRV infection. The expression of each sample was calculated from the control mock-inoculated plant at points 2, 4, 6, and 8 dpi; *—BRV-resistant genotypes.

The quantitative expression profiles of the *pathogenesis-related* 1 (*PR*1) gene after BRV infection were compared in both resistant and susceptible genotypes for treatment periods 2, 4, 6, and 8 dpi (Figure 7). We established that *PR1* was rapidly induced after BRV infection in *Ribes* spp. The expression levels were significantly higher in all genotypes in comparison with the control plants during the entire period of inoculation. The highest expression values of *PR1* were presented in virus-susceptible genotypes *R. nigrum* cvs. Ben Tirran and Vernisaz at 4- and 8-days post-inoculation. Relative *PR1* expression confirms successful virus infection in all microshoots in vitro; gene response to biotic stress was found in all treatments after using our proposed in vitro inoculation method through roots.

## 3. Discussion

A sensitive plant-testing system is an important tool for targeted and accelerated selection of resistant genotypes in *Ribes* breeding programs. The inoculation method under in vitro conditions is appropriate to screen genotypes for their responses to pathogens and could be a useful tool for better understanding the virus–host interaction and resistance mechanisms. Virus inoculation systems suitable for in vitro conditions have been developed in herbaceous plants such as potatoes, lettuce, and tomatoes [24,25,26]. However, inoculation systems for woody perennial plants under in vitro conditions are unexplored. In general, an in vitro system for orchard and berry cultures is usually used for virus elimination and micropropagation [27].

BRV is one of the most serious pathogens in blackcurrant plantations; its control is complicated and the consequences are economically damaging [28]. Heterogenic infection of BRV in the same host plant of field-grown cv. Gojai was shown by the diversity of the sequences (Table 1) and the data of phylogenetic analysis (Figure 1). In this study, we found that multiple infections of BRV with different origins could be detected in one host plant. The natural vector transmitting BRV among bushes is the gall mite. Under controlled conditions, transmission assays showed that 3 h of mite feeding on blackcurrant seedlings was sufficient for virus transmission [21]. However, previous studies have shown that using the gall mite infection method in vitro had difficulties in controlling both the infection pressure and the influence of environmental conditions on the spread of mites and BRV [28,29].

The BRV genome consists of two molecules of positive-sense RNAs: RNA1 and RNA2, which are packed in viral particles. BRV particles are composed of two capsid proteins (54 and 55 kDa) and can replicate in a low range of hosts [22,30]. The 3′ NTRs of BRV are highly similar and the most stable parts of the genome, and are therefore used for detection by PCR [15,31]. Genetic identity among isolates, from 94.6 to 99.6% in the 3′ nontranslated region of RNA2, was also found in our study (Table 1). Only virus particles formed from the 54 kDa fragment can be used for indicator plant infection by mechanical inoculation, but the resulting progeny viruses contain both forms [32]. For the first time, blackcurrant reversion virus was successfully mechanically transmitted from symptomatic blackcurrant leaves to *Chenopodium quinoa* [22] and to other herbaceous plants, *Nicotiana occidentalis* and *Nicotiana tabacum* [17]. These cases of mechanical inoculation are the only two reported in the literature where the virus was transmitted from blackcurrants to herbaceous plants, despite the attempts of scientists to repeat it. Despite these difficulties, sap from *C. quinoa* symptom-bearing leaves was transmitted from 4 to 11 days using carborundum to other herbaceous plants: *C. amaranticolor*, *C. murale*, *N. benthamiana*, *N. clevelandii*, *N. debnyi*, and *N. occidentalis* [16,22]. The successful slash inoculation of young tissue-culture-propagated blackcurrants with the isolated virus from *C. quinoa* was performed by A. Lemmetty and K. Lehto [23]. This plant material is more sensitive due to its soft tissues and low content of phenolic compounds. The first symptoms on the leaves were observed after 5–7 months. Furthermore, the visual symptoms of the disease appeared only after 4–5 years when inoculating blackcurrant woody plants. Additional fulfilling of Koch’s postulates was achieved when mechanically inoculated plants developed typical BRD symptoms on leaves and flower buds [15,18].

It was observed that some plant viruses are difficult to inoculate mechanically because they are transmitted by arthropods during feeding and their localisation to vascular tissue (usually phloem) is limited [33]. The BRV can reach low titres at the beginning of the spread of the infection in the *Ribes* plants, and does not give a titre in genetically resistant plants under natural conditions. Specific antibodies for BRV proteins are not commercially available [10,20]; therefore, polymerase chain reaction is the most sensitive and reliable detection method at present. Using PCR in this study, BRV infection was approved until 8 dpi in BRV-resistant plants *R. dikuscha*, cv. Aldoniai (*R. dikuscha* in progeny) [34] and cv. Ben Gairn (*R. uva-crispa* in progeny) [5].

The amount of virus in the inoculum is an important factor for mechanical infection transmission. Too high or too low virus concentration in the inoculum leads to unsuccessful plant infection [35]. For example, wheat streak mosaic virus (WSMV) inoculum with 2.21 × 10^6^ concentration was most suitable for mechanical inoculation in wheat [36]. In this study, for inoculum preparation we mixed the inoculation buffer with plant sap and triple BRV infection (approximately 2.24 × 10^4^ copies in µL) (Figure 3). All three isolates of BRV (Figure 1) appeared infectious according to the homology of 481 bp fragment (Figure 5) sequencing in this study. 

*Pathogenesis-related 1* gene is the most abundant gene family of *PRs*, and the expression of *PR*1 is used as a molecular marker to indicate plant defence response to different types of insects and pathogens [37]. In our previous study, the biotic stress defence response *PR*1 homolog emphasizing the *R. nigrum* genome was found, and the primer pair identifying this gene was designed [38]. Therefore, in this work, RT-PCR was used to establish the host-plant defence response to viral infection, as biotic stress, for showing the success of the suggested inoculation method (Figure 7). We determined the expression profiles of *PR*1 in resistant and susceptible genotypes during the entire period of the experiment after mechanical BRV inoculation in vitro. The defence response pathway with the determined *PR*1 expression was characteristic for all genotypes, regardless of their resistance to BRV. The expression levels of *PR*1 in susceptible genotypes *R. nigrum* cvs. Ben Tirran and Vernisaz were higher compared to susceptible *R. aureum* and resistant genotypes. However, all susceptible genotypes had a similar tendency showing significant increase in *PR*1 at 4 and 8 dpi. It was found that in the *Ribes* spp., as in other plants, *PR*s are necessary for the establishment of systemic acquired resistance (SAR) in tissues distant from the primary infection site [39].

The in vitro inoculation technique described here is the first reported in the literature where the BRV was successfully transmitted from blackcurrants to blackcurrants with maximum reliability. Several virus transmission methods (leaf damage with carborundum powder; stem soaking in the inoculum; inoculum dripping with syringe into the stem) have been tested on microshoots during our research work, but all of them have failed. Possibilities to successful viral infection through inoculation of *Ribes* plants grown in vitro by using a mechanical approach are possible when the appropriate conditions are used (Figure 4). Rooted microshoots were the most suitable plant material for easy virus entry and movement in the plant because of its soft tissues and low content of phenolic compounds. Based on studies by P. Susi [10] and our data (Figure 2), cold storage (4 °C) in the first period after inoculation is necessary to ensure virus viability and for causing additional abiotic stress in the plant.

It is suggested that the procedure described in this study may be widely applicable for blackcurrant reversion virus mechanical inoculation using sap from *R. nigrum*. The principle for the inoculation method depends on soaking the roots of microshoots in the PBS buffer containing an infectious BRV isolate. The method was used successfully to inoculate susceptible *Ribes* spp. plants with BRV and to study the genetic response for BRV in resistance plants.

## 4. Materials and Methods

### 4.1. Plant Material

Leaves of *R. nigrum* cv. Gojai with symptoms of BRD presented in field conditions were used for preparation of inoculum. Inoculum containing BRV was tested for virus stability, infectivity, and genetic analysis. Rooted microshoots of the species *R. aureum*, *R. dikuscha* and *R. nigrum* cvs. Aldoniai, Ben Gairn, Ben Tirran, Vernisaz were used for inoculation treatments under in vitro conditions.

### 4.2. In Vitro Culture of Ribes spp.

All manipulations with plants in the in vitro system were performed using modified MS medium (Table S1 in Supplementary Materials) [40]. Microshoots of *Ribes* species and cultivars were propagated on medium I (3 weeks), grown on medium II up to 3–4 cm (2 weeks), and rooted on nutrition medium III (4 weeks). Plants grown in this way were used in the inoculation assay in vitro. Micropropagation and rooting of *Ribes* were carried out under controlled conditions in a growth chamber at 21 ± 2 °C, 50–150 µmol m^–2^ s^–1^ light intensity, and 16/8 h photoperiod.

### 4.3. Preparation of Inoculum

The inoculum was prepared from 3.0 g of ground symptomatic blackcurrant cv. Gojai leaves and 10.0 mL of 0.1 M phosphate buffered saline (PBS) buffer (pH 7.5) enriched with 2.0% polyvinylpyrrolidone (PVP), 0.2% sodium sulphite (Na_2_SO_3_), 0.08% 2-mercaptoethanol, and 0.2% Tween 20. The inoculum was filtered through 0.22 µm pore-size sterile filters for microorganism contamination.

### 4.4. Evaluation of BRV Stability in the Inoculum

Aliquots of the prepared inoculum were stored at 4 and 21 °C for 8 days to assess virus stability. RNA was extracted from each treatment of inoculums (fresh and after 1, 2, 4, 6, and 8 days). Virus RNA was evaluated by the PCR method using 3 primer pairs from different sites of the RNA2 3′ NTR regions in the BRV genome (Table 2).

**Table 2:** Primer pairs used for BRV detection and sequencing.

Primer	Sequence 5′ to 3′	Annealing T, °C	Length, bp	Purpose in Research	Reference
P1/P2	GTAATACGCTGGTGTCTC/ GAAAGGACATTTCAGCTC	49	215	For detection of RNA2 plants	[15]
P5/P6	AAACCAGACCCAGGTGAGTG/GGACACTTCCATATAAGTCGGC	60	481	For detection of RNA2 in plants and inoculum	[31]
BCP11/P6	ATTTCGAGCTGTATGGTCG/CTCGGAAGCAGTAGACCT	51	787	For detection of RNA2 in inoculum	[15]
BCP11/P2	ATTTCGAGCTGTATGGTCG/GAAAGGACATTTCAGACTC	51	~1449	For sequencing and genetic analysis of RNA2	[15]

### 4.5. Total RNA Isolation and cDNA Synthesis

Total RNA was isolated from 100.0 µL of inoculum or 0.1 g fresh tissue of microshoots. Plant samples were homogenized in liquid nitrogen. For extraction of total RNA, we used a GeneJET Plant RNA Purification Mini Kit (Thermo Scientific, Vilnius, Lithuania) according to manufacturer’s protocol. Total RNA was used directly as a template for cDNA synthesis with a Maxima H Minus First Strand cDNA Synthesis Kit (Thermo Scientific, Vilnius, Lithuania), and oligo d(T)_20_ primer was used in the reaction.

### 4.6. BRV Detection by PCR

The detection of BRV was carried out by polymerase chain reaction (PCR) in the inoculum and in the *Ribes* spp. microshoots after mechanical inoculation in vitro. BRV-specific oligonucleotide primers and their characteristics are presented in Table 2. PCR reaction was performed in 20.0 µL reaction volume consisting of 11.1 µL H_2_O, 2.5 µL 10× *Taq* buffer + (NH_4_)_2_SO_4_ + MgCl_2_, 2.0 µL 25 mM MgCl_2_, 2.0 µL 2 mM dNTP mix, 0.1 µL of each 0.1 µM forward and reverse primers, 0.2 µL *Taq* DNA polymerase (Thermo Scientific, Vilnius, Lithuania), and 2.0 µL cDNA (about 100 ng). The amplification reaction was performed in a Mastercycler X50a (Eppendorf, Stevenage, UK) under the following conditions: 95 °C for 3 min, 35 cycles at 95 °C for 30 s, 40 s at temperature suitable for primers (Table 2), 72 °C for 40 s, and the final elongation step at 72 °C for 10 min. The amplification products were analysed in 1.5% (*w*/*v*) agarose gel using electrophoresis and visualized by ethidium bromide staining and UV illumination. The size of the PCR products was determined with O’GeneRuler 1kb DNA Ladder (Thermo Scientific, Vilnius, Lithuania).

### 4.7. Purification, Cloning, Sequencing, and Digestion of RNA2 3′ NTR of BRV

Amplified fragments for sequencing with primer pair BCP11/P2 (Table 2) were purified using a GeneJET Gel Extraction Kit (Thermo Scientific, Vilnius, Lithuania). Fragments were ligated into p*JET* 1.2 blunt vector using a CloneJET™ PCR Cloning Kit (Thermo Scientific, Vilnius, Lithuania). The strain *E. coli* JM107 was transformed using TransformAid Bacterial Transformation Kit (Thermo Scientific, Vilnius, Lithuania). Ten samples of plasmids with BRV insertions were sequenced using a Big Dye Terminator v 3.1 Cycle Sequencing Kit and performed on a 3130 Genetic Analyzer (Applied Biosystem, Waltham, MA, USA).

### 4.8. Plant Inoculation In Vitro

Before inoculation, virus-free rooted microshoots were placed in the dark for 48 h at 21 ± 2 °C. Under sterile conditions, roots of 4-week-old microshoots were shortened with a scalpel to a length of 4 mm and soaked for 2 min in sterile tubes with fresh inoculum containing BRV (Figure 4). Inoculated plants were replanted on nutrition medium II (Table S1 in Supplementary Materials) and cultivated for 2 weeks at 4 °C, 16/8 h photoperiod. Mock-inoculated microshoots in PBS buffer were used as the negative control. For RNA isolation, 20 microshoots of each genotype were frozen in liquid nitrogen after cutting their roots and immediately used for RNA isolation.

### 4.9. Gene Expression Analysis in Inoculated Ribes Microplants

Changes in the putative *PR1* gene in mRNA of inoculated blackcurrant genotypes in vitro were analysed using quantitative real-time RT-PCR (qPCR). Reaction was performed in a 11.0 µL reaction volume containing 5.95 μL H_2_O, 1.25 μL 10× *Taq* buffer + (NH_4_)_2_SO_4_ + MgCl_2_, 1.0 μL 2 mM dNTP mix, 1.0 μL 25 mM MgCl_2_, 1.0 µL cDNA, 0.1 μL *Taq* DNA polymerase (Thermo Scientific, Vilnius, Lithuania), 1.0 μL 20× EvaGreen dye (Biotium, Inc., Fremont, CA, USA), and 0.1 μL of each forward and reverse primer [38]. The analysis was carried out on three biological replicates using a Realplex RT-PCR cycler (Eppendorf, Stevenage, UK) under following conditions: 95 °C for 3 min, 35 cycles at 95 °C for 30 s, 55 °C for 40 s, and 72 °C for 40 s; melting point of PCR products: 95 °C for 15 s, 60 °C for 15 s, 60–95 °C for 20 min, 95 °C for 15 s, and hold at 4 °C.

### 4.10. Statistical Analysis

Sequences of the three Lithuanian BRV isolates were uploaded into the NCBI database (accession numbers MH891843, MH891844, and MH891845). A percent identity matrix among the 3′ NTR of RNA2 sequences of BRV was created by the Clustal 2.1 program. The phylogenetic tree was constructed comparing 3 Lithuanian BRV sequences with 21 sequences of homologous region of BRV with reference to the NCBI database. Phylogenetic analysis was performed using the maximum likelihood method implemented in the PhyML program; a bootstrap analysis with 100 replications was performed [41]. Relative expression was assessed by the 2(-Delta Delta C (T)) method [42]. An actin gene, having constant expression levels (data not shown), was used to normalize raw data and calculate relative transcript levels. Means and SEM (standard error of the mean) from independent experiments were subjected to STAT-ENG.

## Figures and Tables

**Table 1:** Percent identity of BRV isolates at nucleotide level of RNA2 3′ NTR regions found in Lithuanian cv. Gojai.

Identity, %	BRV_3-18_LT	BRV_1-18_LT	BRV_7-18_LT
BRV_3-18_LT	*****	94.64	94.64
BRV_1-18_LT		*****	99.59
BRV_7-18_LT			*****

*****—100% identity.

## Data Availability

Not applicable.

## Supplementary Materials

The following supporting information can be downloaded at: https://www.mdpi.com/article/10.3390/plants11131635/s1, Table S1: Composition of modified MS [35] nutrition medium for propagation, growth, and rooting of blackcurrant microshoots, Table S2: Accession numbers and details of blackcurrant BRV sequences obtained from the NCBI database and included in the phylogenetic tree of this study.
