# Supplementary material for: An Effective Method of Ribes spp. Inoculation with Blackcurrant Reversion Virus under In Vitro Conditions
Source: Plants (Basel). 2022 Jun 21;11(13):1635. doi: 10.3390/plants11131635 (PMC9269295; doi:10.3390/plants11131635)
Supplement: Supplementary file 1 [file plants-11-01635-s001.zip › Supplementary files.pdf]

Table S1. Composition of modified MS [35] nutrition medium for propagation, growth and rooting of blackcurrant microshoots

Components	Concentration of the solution	Composition of the propagation medium	Composition of the growth medium	Composition of the rooting medium
	mg l^{-1}	1 l	1 l	1 l
**Macro elements:**		50 ml	50 ml	25 ml
$CaCl_2$	332.02			
KH_2PO_4	170.00			
KNO_3	1900.00			
$MgSO_4$	180.54			
KH_2PO_4	170.00			
**Micro elements:**		2 ml	2 ml	1 ml
$Na_2MoO_4 \cdot 2H_2O$	0.25			
$MnSO_4 \cdot H_2O$	16.90			
$ZnSO_4 \cdot 7H_2O$	8.60			
H_3BO_3	6.20			
KI	0.83			
$CuSO_4 \cdot 5H_2O$	0.025			
$CoCl_2 \cdot 6H_2O$	0.025			
FeNaEDTA	36.70	5 ml	5 ml	2.5 ml
**Vitamins:**				
Myo-inositol	100.00	100 mg	100 mg	50.0 mg
Nicotine acid	250	2 ml	2 ml	1 ml
Piridoxine HCl	250	2 ml	2 ml	1 ml
Thiamine HCl	250	2 ml	2 ml	1 ml
**Others:**				
BAP	250	3 ml	0.5 ml	-
Giberelin	100	-	5 ml	-
Agar		7 g	7 g	7 g
Sucrose		30 g	30 g	30 g

Table S2. Accession numbers and details of blackcurrant BRV sequences obtained from NCBI database and included in the phylogenetic tree of this study

Accession number	Host species or genotype	Symptoms	Origin	References
AF321564	cv. Sunderbyn	Reverted plant	Sweden	[16]
AF321566	P9/5/1	R-form	Finland	
AF321567	cv. Yubileinaya	Reverted plant	Russia	
AF321568	Line 1873–01E1	E-form	Canada/Scotland	
AF321569	cv. Bona	R-form	Poland	
AF321570	cv. Bona	R-form	Poland	
AF321572	Unknown	R-form	Finland	
DQ450978	cv. Viola	R-form	Czech Republic	[17]
DQ450980	cv. Black Smith	R-form	Czech Republic	
DQ450981	cv. Black Smith	R-form	Czech Republic	
DQ450983	cv. Black Smith	R-form	Czech Republic	
DQ450984	cv. Karlštejnský dlouhohrozen	R-form	Czech Republic	
DQ450986	cv. Karlštejnský dlouhohrozen	R-form	Czech Republic	
NC_003502	Labhost *Chenopodium quinoa*)/blackcurrant P9/5/1	R-form	Finland	[13]
KU556775	cv. Zagadka	Galled buds	Russia	[6]
KU556778	cv. Marmoratum	Galled buds	Russia	
KU556784	cv. Stor Klas	Galled buds	Sweden	
KU556779	cv. Titania	No galls	Sweden	
KU556780	cv. Detskoselskaya	Galled buds	Russia	
KU556766	cv. Pamyat Vavilova	Galled buds	Russia	
KU556771	cv. Ben Tirran	Galled buds	Scotland	
